# Molecular Evolution of GII.P31/GII.4_Sydney_2012 Norovirus over a Decade in a Clinic in Japan

**DOI:** 10.3390/ijms25073619

**Published:** 2024-03-23

**Authors:** Hiroshi Ushijima, Sheikh Ariful Hoque, Yuki Akari, Ngan Thi Kim Pham, Tung Phan, Shuichi Nishimura, Masaaki Kobayashi, Kumiko Sugita, Shoko Okitsu, Satoshi Komoto, Aksara Thongprachum, Pattara Khamrin, Niwat Maneekarn, Satoshi Hayakawa

**Affiliations:** 1 Division of Microbiology, Department of Pathology and Microbiology, Nihon University School of Medicine, Itabashi, Tokyo 173-8610, Japan; 2 Cell and Tissue Culture Laboratory, Centre for Advanced Research in Sciences (CARS), University of Dhaka, Dhaka 1000, Bangladesh; 3 Department of Virology, Fujita Health University School of Medicine, Toyoake, Aichi 470-1192, Japan; 4 College of Industrial Technology, Nihon University, Narashino, Chiba 275-8575, Japan; kimnganak@gmail.com; 5 Department of Pathology, University of Pittsburgh, Pittsburgh, PA 15213, USA; 6 Nishimura Pediatric Clinic, Maizuru, Kyoto 625-0036, Japan; 7 Kobayashi Pediatric Clinic, Fujieda, Shizuoka 426-0067, Japan; 8 Sugita Children Clinic, Ibaraki, Osaka 567-0035, Japan; 9 Center for Infectious Disease Research, Research Promotion Headquarters, Fujita Health University, Toyoake, Aichi 470-1192, Japan; 10 Division of One Health, Research Center for GLOBAL and LOCAL Infectious Diseases, Oita University, Yufu, Oita 879-5593, Japan; 11 Faculty of Public Health, Chiang Mai University, Chiang Mai 50200, Thailand; 12 Department of Microbiology, Faculty of Medicine and Emerging and Re-Emerging Diarrheal Viruses Research Center, Chiang Mai University, Chiang Mai 50200, Thailand

**Keywords:** norovirus, GII.P31/GII.4_Sydney_2012, epidemics, ORFs, molecular evolution, deduced amino acid sequences, phylogenetic analysis

## Abstract

Norovirus (NoV) genogroup II, polymerase type P31, capsid genotype 4, Sydney_2012 variant (GII.P31/GII.4_Sydney_2012) has been circulating at high levels for over a decade, raising the question of whether this strain is undergoing molecular alterations without demonstrating a substantial phylogenetic difference. Here, we applied next-generation sequencing to learn more about the genetic diversity of 14 GII.P31/GII.4_Sydney_2012 strains that caused epidemics in a specific region of Japan, with 12 from Kyoto and 2 from Shizuoka, between 2012 and 2022, with an emphasis on amino acid (aa) differences in all three ORFs. We found numerous notable aa alterations in antigenic locations in the capsid region (ORF2) as well as in other ORFs. In all three ORFs, earlier strains (2013–2016) remained phylogenetically distinct from later strains (2019–2022). This research is expected to shed light on the evolutionary properties of dominating GII.P31/GII.4_Sydney_2012 strains, which could provide useful information for viral diarrhea prevention and treatment.

## 1. Introduction

Norovirus (NoV) has remained the leading cause of acute gastroenteritis in people of all ages for several decades [1]. In particular, after the introduction of rotavirus (RV) vaccines, NoV has become the primary cause of acute gastroenteritis (AGE) in children in many countries [2]. Every year, NoV is predicted to cause 699 million illnesses and 219,000 deaths worldwide [1,3]. This small, non-enveloped, single-stranded, positive-sense, RNA virus of the Caliciviridae family demonstrates extensive genetic diversity [4]. Its ~7.5 kb long RNA genome is organized into three open reading frames, ORF1, ORF2, and ORF3, encoding the nonstructural proteins (e.g., VPg, protease, and polymerase), the major capsid protein (VP1), and the minor capsid protein (VP2), respectively [5]. Of these, VP1 possesses the immunodominant antigenic sites: the hypervariable P2 subdomain induces the majority of the blocking antibody responses, whereas antibodies against the less variable P1 and shell domains are more cross-reactive and do not block [6]. Based on the variations in VP1 amino acid (aa) sequences, NoVs are classified into at least 10 genogroups (GI-GX) and 49 genotypes [7]. Among these, genogroup II genotype 4 (NoV GII.4) has been the most common since the mid-1990s, accounting for 62–80% of all NoV outbreaks worldwide over the last two decades [8]. The predominance of NoV GII.4 is associated with the chronological emergence of phylogenetically distinct variants at 2–3-year intervals that are antigenically different due to differences in aa at antigenic sites (A-I) located on the VP1’s outermost surface, allowing escape from previous infections [9]. Since the mid-1990s, GII.4 has caused six major pandemics, including Grimsby_1995, Farmington Hills_2002, Hunter_2004, Den Haag_2006b, New Orleans_2009, and Sydney_2012, and many epidemics, such as Lanzhou_2002, Sakai_2003, Yerseke_2006a, Osaka_2007, Apeldoorn_2007, and HongKong_2019 [9]. Among these, the Sydney_2012 variant appeared with a completely distinct collection of nonstructural polymerase proteins P31 (once known as Pe) that soon outcompeted all others, while still producing a maximum number of cases [10]. This variant further acquired a new P16 polymerase protein, most likely through recombination with the GII.2 [P16] viruses, and produced GII.P16/GII.4_Sydney_2012 strains, which swiftly predominated alongside GII.P31/GII.4_Sydney_2012 worldwide since 2015 [11].

Despite the fact that most GII.4 variants circulated for 2 to 4 years, Grimsby_1995 predominated for more than 8 years, while GII.P31/GII.4_Sydney_2012 has been dominant for more than a decade [9]. Several investigations have focused on the key antigenic sites, namely A-G, and have shown that aa alterations in these epitopes are critical in escaping immune system action, resulting in global epidemics, as seen in the GII.4_Sydney_2012 lineage [12]. However, advances in genome sequencing technologies over the last decade revealed several antigenic changes in major antigenic sites in VP1, despite the fact that the total antigenicity of Sydney_2012 has remained very similar for over a decade, suggesting that the prevalence of GII.4 is not simply due to the antigenic diversity of the capsid protein; nonetheless, comprehensive sequencing is required to understand this further [9]. Several studies have focused on the whole genome sequences of GII.4_Sydney_2012, but only a handful have examined the evolutionary trend of GII.4 Sydney_2012 outbreak strains across time in a particular region. In this study, we analyzed 14 full genomes of GII.P31/GII.4_Sydney_2012 strains collected from 2012 to 2022, mostly from Kyoto, Japan, during epidemics, to better understand the evolutionary characteristics that lead to molecular changes over time, which may aid in the development of effective vaccines against this disease.

## 2. Results

### 2.1. Sample Characteristics

To gain insights into the evolutionary trend of globally dominating NoV strains, 14 NoV GII.P31/GII.4_Sydney_2012-positive stool samples were selected, of which 12 were collected from a single pediatric outpatient clinic in Kyoto prefecture between 2012 and 2022, while the remaining 2 were collected from Shizuoka prefecture in 2022. Full-length genome sequences (~7500 nt) were obtained from all 14 samples, of which 13 remained outbreak strains (Table 1). An outbreak strain was chosen if several children (eight or more) in the clinic were found to be sick with NoV GII.P31/GII.4_Sydney_2012 at the same time. Notably, outbreak strains used in this study were primarily selected from three major outbreaks in Kyoto, each of which lasted for several months. To comprehend the genetic alterations during a single epidemic, several samples from both nearby and distant samples were chosen from each epidemic. The children were aged from 7 to 40 months. Co-infections were detected in four children with classic Astrovirus 1 (AstV1) in both Kyoto and Shizuoka in 2021–2022.

### 2.2. Phylogenetic Analysis and Nucleotide (nt) Identities of Individual ORFs

**Figure 1:**
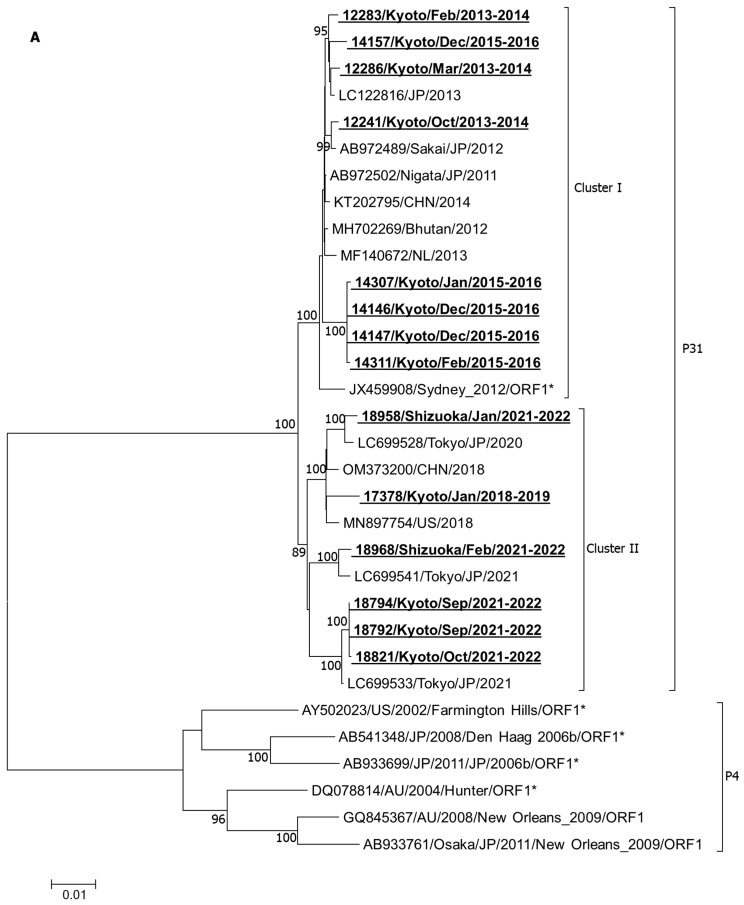

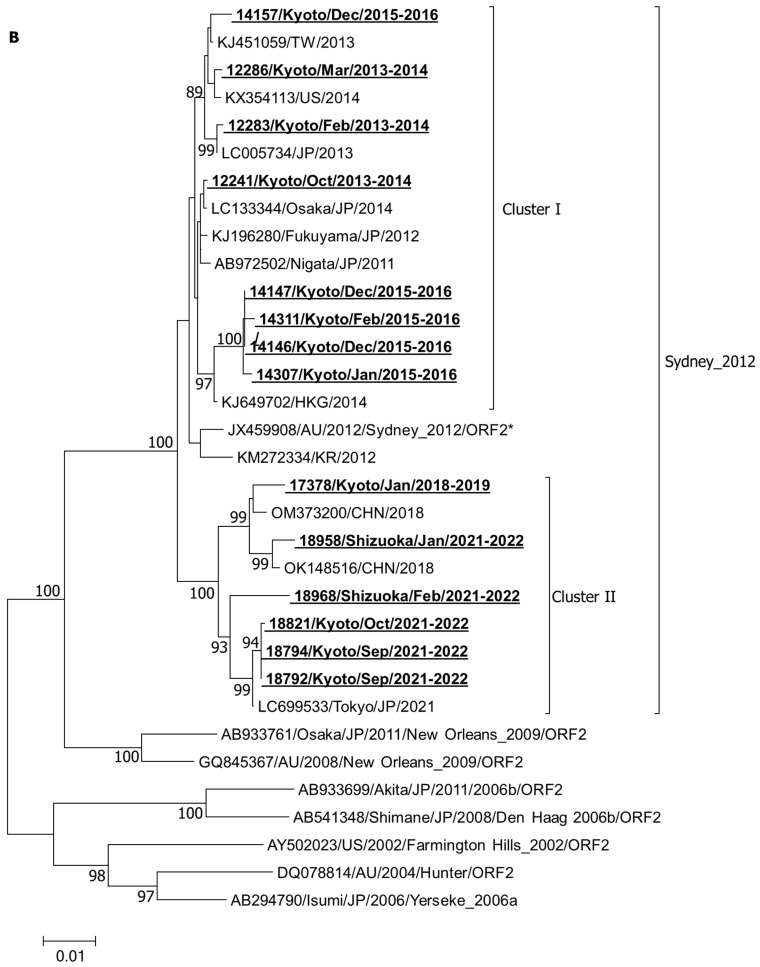

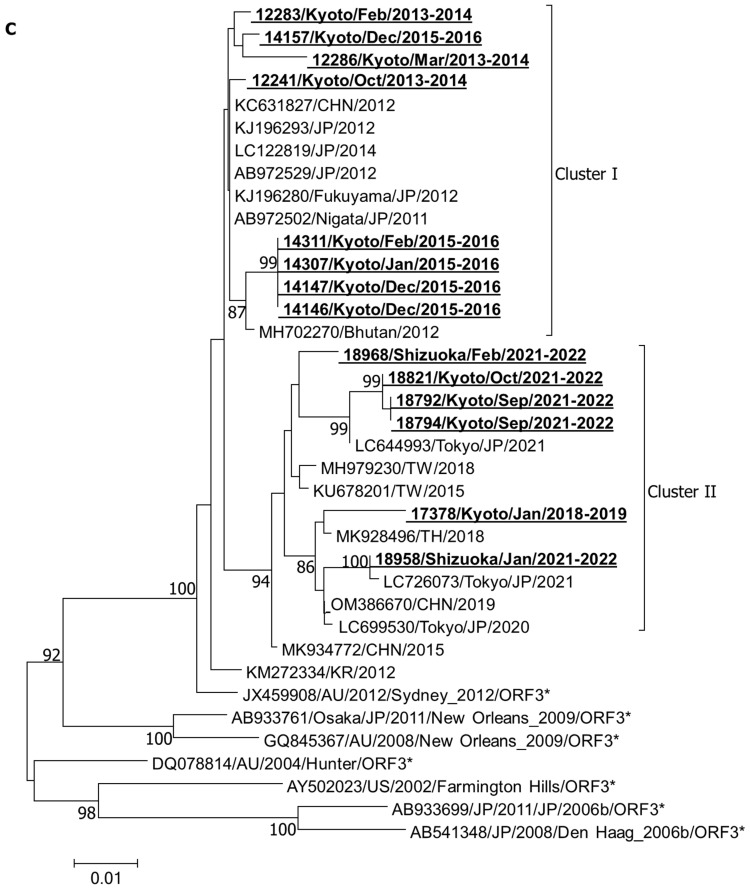
Phylogenetic analysis of ORF1 (**A**), ORF2 (**B**), and ORF3 (**C**). These trees were constructed by means of the neighbor-joining method with the Kimura 2-parameter model nucleotide substitution model. The statistical significance was tested using 1000 bootstrapping replicates and values ≥ 80% are shown at the branch nodes. The strains detected in this study are shown in bold with underlining. Asterisks represent the prototypes.

All three phylogenetic analyses of individual ORFs, including ORF1 (Figure 1A), ORF2 (Figure 1B), and ORF3 (Figure 1C), showed that all the analyzed strains belonged to the GII.P31/GII.4_Sydney_2012 genotype, showing less association with other major GII.4 variants like Farmington_Hills_2002, Hunter_2004, Yerseke_2006a, Den Haag_2006b, and New Orleans_2009. Importantly, in all three ORFs, the analyzed strains remained divided into two clusters within the GII.P31/GII.4_Sydney_2012 lineage. Namely, the earlier strains from 2013 to 2016 remained associated with the strains from a similar time in cluster I, while the later strains from 2019 to 2022 and their associates remained clustered into cluster II, suggesting that the genetic make-up of all three ORFs gradually changed over time.

Interestingly, although the studied strains belonged to the GII.P31/GII.4_Sydney_2012 genotype, none of these strains demonstrated close association with the original GII.P31/GII.4_Sydney_2012 prototype (JX459908); rather, they remained more closely related to the AB972502 strain, another GII.P31/GII.4_Sydney_2012 variant, isolated in 2011 in Niigata (AB972502) (Table 2). The average nt identities in ORF1, ORF2, and ORF3 were determined to be 98.9%, 98.4%, and 98.2% with the JX459908 strain, while they were 99.5%, 99.0%, and 99.3% with the AB972502 strain, respectively, for earlier strains. Meanwhile, for later strains, these nt identities were decreased similarly (0.6–1.1%) from both reference strains. Together, our data reveal that our strains remained genetically closer to the Japanese Sydney_2012 variant (AB972502) than the original Sydney_2012 prototype (JX459908) and additional genetic evolution occurred in later strains.

### 2.3. Comparative Analysis of the ORF1

The 5100 nt long ORF1 region of NoV GII represents the major nonstructural proteins including p48, nucleoside-triphosphatase (NTPase), p22, VPg, protease, and the RNA-dependent RNA polymerase (RdRp) of about 1700 aa long. A comparative analysis of the deduced aa sequences of the ORF1 with that of the prototype strain GII.P31/GII.4_Sydney_2012 (JX459908) demonstrated 38 (2.2%) aa changes, of which 18 remained informative (detected in >1 strains). Among them, six aa changes, including L29F, K497R, S763G, I784T, S1044G, and S1346N, remained common in other NoV GII.4_Sydney_2012 strains, including the AB972502 (Table 3). Maximum informative aa changes in ORF1 in later strains were observed in RdRp (K1368R, K1547R, I1648V, and I1653V), followed by p22 (I711V and S774N) and p48 (S90F). In addition, some informative changes (K779R and T875A in p22, and M1315K in RdRp) were found in earlier strains but not in later strains.

### 2.4. Comparative Analysis of the ORF2

The 1623 nt long ORF2 encodes the major structural capsid protein VP1 of 541 aa residues, on which S (41–213 aa), P1 (222–275 aa and 419–540 aa), and P2 (276–418 aa) domains are segmented. In ORF2, 24 (4.4%) variable sites were observed, of which 15 (2.7%) were informative. Among these, four aa changes (N309S, D310N, H414P, and V540L) from JX459908 remained very common in other strains including AB972502 (Table 4). Several aa changes have been observed in the P2 domain that differentiate earlier strains from later strains, including non-epidemic strains, and those isolated from Shizuoka, like T285A, R297H, V333M, D372N, and R373N, were observed mainly in later strains, while T340A, R373H, and G393S were observed in earlier strains only. T534A in the P1 domain was observed in later strains only. Informative aa variations were identified in major antigenic sites [9], namely A (at 297, 372, and 373), C (at 340 and 377), D (at 393), and E (at 414), as well as in the variable motifs B (at 333) and H (at 309 and 310). The antigenic sites G and I remained conserved in earlier reports [9]. However, no substitution was observed in histo-blood group antigens (HBGAs) binding pocket sites I (aa 343–347), II (aa 374), and III (aa 442–443) [13].

### 2.5. Comparative Analysis of the ORF3

An 807 nt long ORF3 encodes the minor capsid protein VP2 of 269 aa residues. Here, aa changes were found in 26 (9.6%) residues, of which 15 (5.5%) remained informative. Among these, K73R, F92S, V183G, S211F, and V241I were common in many strains, but not in JX459908 (Table 5). A4T, I101T, T164A, T174I, and the deletion of 145–146 residues remained common in later strains, while S112N, P141L, and G247S remained common in earlier strains.

## 3. Discussion

This study aimed to investigate the evolutionary changes in the genetics of the globally predominant GII.P31/GII.4_Sydney_2012 variant in a single community from 2012 to 2022. This GII.P31/GII.4_Sydney_2012 variant grew and predominated mostly after the introduction of RV vaccines. In fact, RV vaccines continued to reduce disease severity in RV illness [14,15,16,17] but failed to regulate the diversity in RV genotypes [18,19,20,21,22] and the increasing trends of other diarrheal viruses including NoV [23]. However, we need to investigate carefully how viral gastroenteritis will change after the COVID-19 pandemic and the spread of RV vaccination. AGE viruses persisted in substantial numbers in environmental samples even throughout the COVID-19 pandemic [24,25,26,27]. Therefore, to control the overall burden of childhood diarrhea, it is important to examine the evolutionary changes in this dominant strain along with the adoption of RV vaccines.

This GII.P31/GII.4_Sydney_2012 variant remained involved in more than six outbreaks in the Kyoto region during this period. We chose samples from the three main outbreaks that each continued for several months in Kyoto, along with one non-epidemic strain (17378). We considered that the possibility of genetic evolution during the outbreak period may have contributed to extending the duration of the outbreak. In this regard, samples from both adjacent and distant times were chosen from each outbreak. In addition, two strains from the Shizuoka outbreak of 2022 were investigated for comparison. This GII.4_Sydney_2012 genotype prevailed in the Kyoto region during this period. Although the RdRp genotype was not always examined, the recombinant GII.P16/GII.4_Sydney_2012 genotype was detected rarely in Kyoto and not in any epidemic. Other NoV GII genotypes that were detected in Kyoto during the 2012–2021 period included GII.2, GII.3, GII.4 (2006b), GII.4 (2008a), II.4 (2008b), GII.4 (2009), GII.6, GII.14 and GII.17. Among these, GII.3 and GII.17 remained involved in outbreaks in Kyoto in 2014 and 2015, respectively.

Although GII.P31/GII.4_Sydney_2012 appeared as a pandemic variant in 2012 reported first in Australia (Sydney-NSW0514/2012/AU accession JX459908) [28], it was also detected in 2010–2011 in several countries, including South Africa [29], Italy [30], the USA [31] and Japan [32,33]. The full genome sequence of a GII.P31/GII.4_Sydney_2012 strain (AB972502) isolated from Niigata, Japan, in 2011 exhibited 99.65%, 98.89%, and 98.13% nt identities in ORF1, ORF2, and ORF3, respectively, with those of the original JX459908 prototype. Interestingly, our strains remained closer to AB972502 rather than the original prototype, JX459908 (Table 2). Little diversity of the JX459908 strain from other Sydney_2012 strains has been also noticed in other studies [33]. All six strains detected between 2018 and 2022 clustered away from the earlier strains in all three ORFs (Figure 1). In fact, strains of each epidemic season changed gradually over the course of time, which remained consistent with previous findings [33]. Interestingly, a similar pattern of phylogenetic distribution was observed for studied strains in all three ORFs, suggesting that all three ORFs remained prone to evolutionary changes.

Importantly, the substitution of aa included many important functional positions. For instance, the substitution of 393 residues of ORF2 may affect HBGA recognition [32]. All of our earlier strains detected before the 2018–2019 season expressed serine at 393 residues, which remained similar to that of the AB972502/Niigata strain as well as many other strains, like KJ649702/Hu/HKG/2014, KJ451059/2013/TW, LC005734/Hu/JP/2013, and AB933761/Osaka/2011/2009. However, later strains detected since 2018–19 expressed Glycine at the 393 position, which remained similar to GII.P31/GII.4_Sydney_2012 prototype (JX459908) as well as KM272334/KR/2012, LC133344/Osaka/2014/JP, KX354113/2014/USA, OM373200/2018/CHN, LC699533/Tokyo/2021, OK148516/Hu/GZ19/2018/CHN, and AB933699/Akita/2011/2006b. While a single aa substitution remained very common, a few positions (such as 90 and 774 at ORF1, 340, 372, and 373 at P2 of ORF2, and 146, 164, and 174 in ORF3) showed two or more aa substitutions, which should be regarded as more vulnerable sites (Table 3, Table 4 and Table 5). Major aa changes remained similar in the same epidemic as well as in the non-epidemic strain 17378/Kyoto/Jan/2018–2019 and strains that were collected from Shizuoka (18958 and 18968/Shizuoka/2021–2022) in the same season. Figure 1 shows that all three ORFs of the Kyoto strains (18792, 18794, 18821) and Shizuoka strains (18958, 18968) of the 2021–2022 epidemic as well as the 17378 non-epidemic strain existed in the same cluster II in phylogenetic analyses, though there were few aa differences in these strains (Table 3, Table 4 and Table 5). In particular, the 17378 non-epidemic strain exhibited a few aa substitutions at positions 67, 161, 842, and 1522 in ORF1, 119 in ORF2, and 13, 134, 137, 159, and 181 in ORF3 that were absent from all epidemic strains. Though the precise function of these aa substitutions is still unknown, it is plausible that this strain cannot become strong enough to start an epidemic as a result of these unusual aa mutations.

Finally, this study presented the evolutionary changes of nucleotides at different residues in GII.P31/GII.4_Sydney_2012 outbreak strains. Several informative mutations were identified, but their role in the phenotype remains unknown. The lack of antigenic testing to comprehend the role of substituted aa and the small sample size drawn non-randomly only from three major epidemics remained the main shortcomings of this study. Nonetheless, this study presents all the mutations of the 14 strains and provides a general understanding of the genetic alterations of the GII.P31/GII.4_Sydney_2012 strains in the same region/season as well as variations over time. This information may be helpful in determining the significance of significant mutations in the future.

## 4. Materials and Methods

### 4.1. Sample Selection and RNA Extraction

As a part of routine screening of diarrheal viruses, stool samples from AGE children were collected under the approval of the ethical committees of the University of Tokyo (1139) and Nihon University (25-13-0, 29-9-0, 29-9-1) and were investigated for the genetic diversity of 11 AGE viruses including NoV GII and 10 other enteric viruses including rotavirus (RV) A, B, and C, NoV GI, sapovirus (SaV), adenovirus (AdV), human astrovirus (AstV), human parechovirus (HPeV), enterovirus (EV), and Aichi virus which were detected using four sets of primers (A, B, C, and D) in four different multiplex RT-PCR reactions, as described previously [34]. NoV GII was further subjected to polymerase-capsid dual typing by means of sequence analysis of the polymerase-capsid junction region as described earlier [35].

Finally, 14 NoV GII.P31/GII.4_Sydney_2012-positive stool samples were selected between 2012 and 2022: 12 from Kyoto and 2 from Shizuoka prefecture pediatric outpatient clinics. For next-generation sequencing (NGS), RNA was extracted from the 10% fecal suspensions using the QIAamp Viral RNA mini kit (QIAGEN, Hilden, Germany) following the manufacturer’s instructions without using carrier RNA.

### 4.2. Next-Generation Sequencing (NGS)

The extracted RNA was subjected to Illumina MiSeq NGS as described previously [36]. In brief, a 200 bp fragment library ligated with bar-coded adapters was constructed for 14 NoV GII strains using an NEBNext Ultra RNA Library Prep Kit for Illumina v 1.2 (New England Biolabs, Ipswich, MA, USA) according to the manufacturer’s instructions. A cDNA library was isolated using Agencourt AMPure XP magnetic beads (Beckman Coulter, Brea, CA, USA). After evaluating the quality and quantity of the isolated cDNA library, 151-cycle paired-ends-read nucleotide sequencing was performed on a MiSeq Reagent Kit v2 (Illumina Inc., San Diego, CA, USA). MiSeq sequence data were analyzed using CLC Genomics Workbench v8.0.1 (CLC Bio, Aarhus, Denmark). Contigs were assembled from the obtained sequence data (trimmed) using de novo assembly. Using the assembled contigs as query sequences, the Basic Local Alignment Search Tool (BLAST) non-redundant nucleotide database was used to determine which contigs represented full-length nucleotide sequences for each gene segment of the 14 NoV strains. To further refine the contigs, sequence reads for each gene segment were mapped back to the assembled contig. The nucleotide sequences of the strains were translated into aa sequences using GENETYX v11 (GENETYX, Tokyo, Japan)

### 4.3. Phylogenetic Analysis

Sequences were segmented into individual ORFs based on the sequences of ORFs of Sydney_2012 prototype (JX459908). Reference sequences were obtained from the NCBI GenBank database (https://blast.ncbi.nlm.nih.gov/Blast.cgi) accessed on 31 January 2024. Phylogenetic trees were constructed after multiple sequence alignments using MEGA7 software using the neighbor-joining method with the Kimura 2-parameter model and statistical significance testing by 1000 bootstrapping replicates. The deduced aa sequences were determined using BioEdit v7.2.5 software. The ORF1 was segmented into p48, NTPase, p22, Vpg, Protease, and RdRp based on their genetic mapping in the strain MK934772. The ORF2 was segmented into NTA, S, P1, and P2 domains as described earlier [5]. The variability of the deduced aa sequences was determined using BioEdit v7.2.5 software.

### 4.4. Nucleotide Accession Number

Whole genome sequences of 14 GII.P31/GII.4_Sydney_2012 strains determined in this study were deposited in the GenBank database for the accession numbers and shown in Table 1.

## 5. Conclusions

This study demonstrated the genetic evolution of GII.P31/GII.4_Sydney_2012 strains that caused epidemics in a place at different times. By enabling researchers to gain a better understanding of gene activity and the mechanisms underlying reinfection, these data will be helpful in the prevention of infection and the creation of efficient vaccinations. Future evolutionary analyses of this globally dominant genotype will likewise need to continue.

## Figures and Tables

**Table 1:** Whole genome sequences of 14 GII.P31/GII.4_Sydney_2012 strains determined in this study were deposited in the GenBank database for the accession numbers.

ID	Place	Collection Date	Age (m)	NoV Genotype	Characteristics	Coinfection	GenBank Accession Number
12241	Kyoto	23 October 2013	14	GII.P31-GII.4	Epidemic	-	OR844370
12283	Kyoto	12 February 2014	16	GII.P31-GII.4	Epidemic	-	OR844371
12286	Kyoto	4 March 2014	7	GII.P31-GII.4	Epidemic	-	OR844372
14146	Kyoto	1 December 2015	18	GII.P31-GII.4	Epidemic	-	OR844373
14147	Kyoto	1 December 2015	23	GII.P31-GII.4	Epidemic	-	OR844374
14157	Kyoto	1 December 2015	18	GII.P31-GII.4	Epidemic	-	OR844375
14307	Kyoto	1 January 2016	22	GII.P31-GII.4	Epidemic	-	OR844376
14311	Kyoto	1 February 2016	10	GII.P31-GII.4	Epidemic	-	OR844377
17378	Kyoto	11 January 2019	21	GII.P31-GII.4	Non-epidemic	-	OR844378
18792	Kyoto	17 September 2021	27	GII.P31-GII.4	Epidemic	Ast1	OR844379
18794	Kyoto	17 September 2021	26	GII.P31-GII.4	Epidemic	Ast1	OR844380
18821	Kyoto	8 October 2021	20	GII.P31-GII.4	Epidemic	Ast1	OR844381
18958	Shizuoka	27 January 2022	9	GII.P31-GII.4	Epidemic	Ast1	OR844382
18968	Shizuoka	10 February 2022	40	GII.P31-GII.4	Epidemic	-	OR844383

**Table 2:** Nucleotide identities of ORFs 1–3 of 14 GII.P31/GII.4_Sydney_2012 strains in this study with reference strains.

Collection Year	ID/Collection Year	Nucleotide Identity					
		ORF1		ORF2		ORF3	
		JX459908	AB972502	JX459908	AB972502	JX459908	AB972502
		99.29		98.95		98.88	
	12241/2013	98.98	99.65	99.01	99.69	98.63	99.75
2013–2016	12283/2014	99.02	99.68	98.71	99.14	98.51	99.63
	12286/2014	99.04	99.63	98.52	99.2	97.76	98.63
	14146/2015	98.8	99.39	98.15	98.83	98.13	99.25
	14147/2015	98.8	99.39	98.15	98.83	98.13	99.25
	14157/2015	98.82	99.49	98.34	99.01	98.38	99.5
	14307/2016	98.72	99.31	98.21	98.64	98.13	99.25
	14311/2016	98.74	99.33	97.97	98.64	98.13	99.25
**Average**		**98.9**	**99.5**	**98.4**	**99.0**	**98.2**	**99.3**
	17378/2019	97.62	98.07	96.79	97.47	95.9	97.01
2019–2022	18792/2021	97.81	98.31	97.29	97.72	95.52	96.64
	18794/2021	97.81	98.31	97.29	97.72	95.52	96.64
	18821/2021	97.77	98.27	97.23	97.66	95.65	96.77
	18958/2022	97.62	98.13	96.73	97.29	96.39	97.51
	18968/2022	97.72	98.27	96.61	97.16	96.89	98.01
**Average**		**97.7**	**98.2**	**97.0**	**97.5**	**96.0**	**97.1**

**Table 3:** Comparison of deduced amino acid sequences of ORF1 of GII.P31/GII.4_Sydney_2012 variants.

ORF1	p48								NTPase				p22													Vpg	Pro			RdRp								
	29	50	67	84	90	118	161	286	389	429	433	497	711	761	763	774	779	783	784	788	842	845	847	850	875	961	1044	1045	1052	1315	1346	1368	1514	1522	1547	1647	1648	1653
JX459908	L	K	K	N	S	S	L	I	K	K	A	K	I	N	S	S	K	A	I	N	K	V	D	E	T	K	S	A	P	M	S	K	V	I	K	K	I	I
12241/Kyoto/Oct/2013–2014	F											R			G				T	S						R	G				N					R		
12283/Kyoto/Feb/2013–2014	F											R			G				T								G				N							
12286/Kyoto/Mar/2013–2014									R			R		S	G				T								G				N							
14146/Kyoto/Dec/2015–2016	F											R			G		R		T						A		G			K	N							
14147/Kyoto/Dec/2015–2016	F											R			G		R		T						A		G			K	N							
14157/Kyoto/Dec/2015–2016	F					I						R			G			V	T			A		D			G				N		I					
14307/Kyoto/Jan/2015–2016	F											R			G		R		T						A		G		T	K	N							
14311/Kyoto/Feb/2015–2016	F	E										R			G		R		T						A		G			K	N							
17378/Kyoto/Jan/2018–2019	F		E	N			F				T	R			G				T		R						G				N			V	R			
18792/Kyoto/Sep/2021–2022	F				F							R	V		G	N			T								G				N	R			R		V	V
18794/Kyoto/Sep/2021–2022	F				F							R	V		G	N			T								G				N	R			R		V	V
18821/Kyoto/Oct/2021–2022	F				F							R	V		G	N			T								G	V			N	R			R		V	V
18958/Shizuoka/Jan/2021–2022	F			N				V		R	T	R			G				T				N				G				N				R			
AB972502/Niigata/JP/2011	F											R			G				T								G				N							
KM272334/KR/2012	F											R				G			T	S							G				N							
KT202795/CHN/2014	F											R			G				T								G				N							
MH702269/Hu/Bhutan/2012	F											R			G				T								G				N							
MF140672/Hu/NL/2013	F				A							R			G				T								G				N							
MN897754/Hu/US/2018	F			N								R			G				T								G				N							

**Table 4:** Comparison of deduced amino acid sequences of ORF2 of GII.P31/GII.4_Sydney_2012 variants.

ORF2	NTA			S		P1		P2														P1		
	8	9	17	98	119	231	244	285	297	309	310	333	340	372	373	377	378	393	395	407	414	460	534	540
JX459908	A	N	N	G	V	V	I	T	R	N	D	V	T	D	R	A	N	G	T	S	H	Y	T	V
12241/Kyoto/Oct/2013–2014										S	N							S	A		P			L
12283/Kyoto/Feb/2013–2014											N				H			S			P			L
12286/Kyoto/Mar/2013–2014		S							H	S	N				H	T		S			P			L
14146/Kyoto/Dec/2015–2016										S	N	M	A		H			S			P			L
14147/Kyoto/Dec/2015–2016										S	N	M	A		H			S			P			L
14157/Kyoto/Dec/2015–2016		S								S	N				H			S			P			L
14307/Kyoto/Jan/2015–2016							V				N	M	A		H			S			P			L
14311/Kyoto/Feb/2015–2016										S	N	M	A		H		I	S			P			L
17378/Kyoto/Jan/2018–2019	V				I				H	S	N	M		N	N						P		A	L
18792/Kyoto/Sep/2021–2022								A	H	S	N	M		N	N						P		A	L
18794/Kyoto/Sep/2021–2022								A	H	S	N	M		N	N						P		A	L
18821/Kyoto/Oct/2021–2022								A	H	S	N	M		N	N						P		A	L
18958/Shizuoka/Jan/2021–2022	V								H	S	N	M		N	N						P		A	L
18968/Shizuoka/Feb/2021–2022			S	S		I			H	S	N	M		S	N	T				G	P	H	A	L
AB972502/Niigata 2011/JP										S	N							S			P			L
KM272334/KR/2012											N				H									
KJ649702/Hu/HKG/2014										S	N	M						S			P			L
KJ451059/2013/TW		S								S	N							S			P			L
LC133344/Osaka/2014/JP										S	N										P			L
LC005734/Hu/JP/2013											N				H			S			P			L
KX354113/2014/USA		S								S	N				H						P			L
OM373200/2018/CHN	V								H	S	N	M		N	N						P		A	L
LC699533/Tokyo/2021								A	H	S	N	M		N	N						P		A	L
OK148516/Hu/GZ19/2018/CHN	V								H	S	N	M		N	N						P		A	L
AB933699/Akita/2011/2006b					I	I					N		G	E	N	T	H				P	H		L
AB933761/Osaka/2011/2009					I						S				N	T		S						L

**Table 5:** Comparison of deduced amino acid sequences of ORF3 of GII.P31/GII.4_Sydney_2012 variants.

ORF3	4	13	23	73	78	82	92	101	112	134	137	141	145	146	149	150	159	164	174	181	183	191	211	241	247	268
JX459908	A	V	N	K	M	K	F	I	S	P	S	P	N	L	A	V	S	T	T	T	V	F	S	V	G	A
12241/Kyoto/Oct/2013–2014				R		R	S														G		F	I		
12283/Kyoto/Feb/2013–2014				R			S		N												G		F	I		
12286/Kyoto/Mar/2013–2014			S	R	V		S		N										A		G	S	F	I	S	
14146/Kyoto/Dec/2015–2016				R			S					L									G		F	I	S	
14147/Kyoto/Dec/2015–2016				R			S					L									G		F	I		
14157/Kyoto/Dec/2015–2016				R			S		N												G		F	I	S	
14307/Kyoto/Jan/2015–2016				R			S					L									G		F	I	S	
14311/Kyoto/Feb/2015–2016				R			S					L									G		F	I		
17378/Kyoto/Jan/2018–2019		I		R			S			S	P			F			F			A	G		F	I		V
18792/Kyoto/Sep/2021–2022	T			R			S	T					-	-				A	I		G		F	I		V
18794/Kyoto/Sep/2021–2022	T			R			S	T					-	-				A	I		G		F	I		V
18821/Kyoto/Oct/2021–2022	T			R			S	T					-	-				A	I		G		F	I		V
18958/Shizuoka/Jan/2021–2022				R			S							F				I	A		G		F	I		V
18968/Shizuoka/Feb/2021–2022				R			S								T	A			I		G		F	I		V
AB972502/Niigata 2011/JP				R			S														G		F	I		
KM272334/KR/2012				R			S														G		F			
LC726073/Tokyo/2021				R			S							F				I	A		G		F	I		V
MH979230/2018/TW				R			S												I		G		F	I		V
MK928496/TH/2018				R			S							F							G		F	I		V
OM386670/CHN/2019				R			S							F							G		F	I		V
MH702270/Hu/Bhutan/2012				R			S														G		F	I		
AB933699/Akita/2011/2006b				R			S							P					V		G		F			V
AB933761/Osaka/2011/2009				R			S														G		F			V

## Data Availability

All data are available within the manuscript.
